# Bacterial nanocellulose films functionalized with Janus nanoparticles: Preparation and application in chicken meat preservation and safety

**DOI:** 10.1038/s41598-026-39029-x

**Published:** 2026-02-06

**Authors:** Negar Alizadeh, Mehran Moradi, Rahim Molaei, Roghayieh Razavi

**Affiliations:** 1 Department of Food Hygiene and Quality Control, Faculty of Veterinary Medicine, Urmia University, Urmia, Iran; 2 Materials Synthesis Laboratory, Carbon Tech Industrial Group, Carbon Tech, Urmia, Iran

**Keywords:** Janus nanoparticles, Bacterial nanocellulose, Active packaging, *Salmonella typhimurium*, Shelf life, Chicken meat, Biotechnology, Chemistry, Materials science, Microbiology, Nanoscience and technology

## Abstract

Janus nanoparticles (JPs) were prepared from hydrophobic carbon dots and carboxymethylcellulose via an ex-situ method, and incorporated into bacterial nanocellulose (BNC) films at concentrations of 0.01%, 0.02%, and 0.03%. Cytotoxicity test revealed a toxic effect of JPs on human gastric cancer cells only at concentrations above 5 mg/mL. Fourier transform infrared spectroscopy confirmed the successful incorporation of JPs without interfering with BNC network. JPs incorporation reduced the tensile strength and elongation at break of the films but improved the radical scavenging activity of BNC in a concentration-dependent manner. The diameters of inhibition zones for BNC films containing 0.01%, 0.02%, and 0.03% JPs against *Salmonella* Typhimurium were 17.1 mm, 20.7 mm, and 27.5 mm, respectively. Upon application to chicken breast meat, all treated samples exhibited an inhibitory effect on *S*. Typhimurium, as indicated by the absence of detectable levels on day 16. For BNC-JPs samples, a notable decrease in mesophilic bacterial counts was observed, representing reductions of 3.2, 4.6, and 5.6 log₁₀ CFU/g at JP 0.01%, 0.02%, and 0.03%, respectively. The BNC-JPs-treated samples also had lower volatile nitrogenous compounds and lipid oxidation levels. These findings highlight the potential of BNC-JPs films as green, active food packaging materials with commercial potential.

## Introduction

Fresh chicken meat is more prone to microbial growth and lipid deterioration during storage due to its high moisture and protein content and mildly alkaline pH. In addition to microbial spoilage, meat can also be contaminated with human pathogens such as *Salmonella* spp., *Campylobacter jejuni*, *Escherichia coli* O157:H7, *and Listeria monocytogenes*^1,2^. Among them, *Salmonella enterica* serovar Typhimurium (*S.* Typhimurium) is of utmost importance because of its high pathogenicity, which frequently leads to contamination or colonization in humans and animals, as well as in the poultry sector, with considerable economic losses^3^. Moreover, diseases caused by this species pose a significant threat to food safety and public health, typically leading to death and expensive medical expenses^4^. Thus, the key function of the poultry sector is to maintain product freshness, extend shelf life, and enhance the product appeal to consumers by efficiently preventing oxidation and spoilage^2^.

Antimicrobial packaging is a form of active packaging that enhances the safety and shelf life of food through the use of antimicrobial agents in synthetic or bio-based polymer matrices^5^. Bacterial nanocellulose (BNC) is a biodegradable and non-toxic polysaccharide polymer derived from certain strains of beneficial bacteria, notably *Komagataeibacter* spp. ^6^. Because of its unmatched biocompatibility, flexibility, biodegradability, water-retention capacity, porosity, barrier effect, and transparency, BNC has gained remarkable interest in various fields, ranging from food and medicine to textiles, cosmetics, and water treatment^7^. The porous three-dimensional nanofibrillar architecture of BNC renders it a suitable substrate for the loading and controlled delivery of active ingredients, such as antimicrobials, antioxidants, oxygen scavengers, and nanoparticles^8^. Incorporating antimicrobial agents into BNC films is of special importance because of the possibility of instant inhibition of microbial growth on food surfaces, which increases food safety and shelf life. Various antimicrobial agents have been successfully incorporated into BNC matrices, such as metal oxides (zinc oxide^9^ and nanoparticles, such as silver (Ag)^9^ and carbon dots (CDs)^10^, with all having varied modes of antimicrobial action. Despite this progress, most current BNC-based systems continue to employ nanomaterials with symmetrical structures and single functionalities, a strategy that may reduce their performance in more complex food systems. Thus, the development of asymmetrical multifunctional nanostructures, such as Janus nanoparticles (JPs), with tunable surface properties and dual functionality, is a potential next-generation approach to overcome these limitations.

JPs are anisotropic particles composed of two or more constituents with different properties. The inherent asymmetry endows them with special optical, electrical, and magnetic properties, along with excellent multifunctionality and tunable surface functionality, making them extremely versatile^11^. For instance, the co-presence of hydrophilic and hydrophobic components enables JPs to display amphiphilic behavior, which can aid in dual-function release or combination therapy^12,13^. Several criteria are employed to classify JPs. Morphologically, they range from simple spheres to more complex forms, such as rods, dumbbells, snowman, discs, fungi, and half-strawberries^14^. They can be classified as nanoparticles or microparticles^15^. They can be inorganic, polymeric, or hybrid. Several fabrication techniques are used in JP synthesis, including self-assembly, phase separation, masking, nucleation and growth, microfluidic synthesis, and primer-directed synthesis^4,11,16^. The choice of fabrication technique typically depends on the characteristics of the materials used.

These multi-compartmental particles are increasingly popular in the food industry, where they are used as emulsifiers, intelligent packaging, biosensors, and delivery agents for the controlled release of drugs or bioactive compounds^13,17^.

Recent studies have demonstrated the usefulness of multifunctional nanostructures in food preservation. Specifically, bifunctional gold–carbon dot–silver nanoclusters allow for ultrasensitive detection and destruction of *Listeria monocytogenes*^18^, and CO_2_-activated labels can diffuse eugenol and prolong fruit shelf life^19^. Numerous Janus-based materials, ranging from antimicrobial absorbent pads^20^ to multifunctional zein/chitosan films^21^ and gradient dual-channel electrospun nanofibers^22^, have demonstrated strong antimicrobial and antioxidant properties, significantly prolonging meat freshness and storage life. Although JP-based active food packaging is promising, studies in this area are still new, and publications remain limited.

Most BNC-based active packaging systems have been developed using single-functional symmetric nanomaterials, resulting in inferior performance for complex food matrices. Current studies seldom offer dual antimicrobial–antioxidant functions, and their ability to control release from BNC scaffolds still needs to be improved. Furthermore, JPs are spherical materials with anisotropic structures and dual actions that have rarely been studied in food packaging and have not yet been applied to BNC matrices. Thus, this study reports the development of a novel bio-based multifunctional active packaging material using structurally anisotropic JPs embedded in BNC matrices. These specially designed JPs feature a hydrophilic hemisphere made of carboxymethyl cellulose (CMC) and a hydrophobic section enriched with CDs, enabling them to perform dual functions via controlled release and surface interaction. By taking advantage of the nanofibrillar structure and high loading capacity of BNC, the inclusion of JPs is expected to enhance the antimicrobial and antioxidant properties of the film through a controlled release mechanism. The composite BNC-JPs films were characterized and evaluated for their potential to inhibit *S.* Typhimurium growth in chicken breast meat, as well as to prevent microbial and chemical spoilage during cold storage. This study marks the first application of JPs in BNC-based packaging systems and provides detailed insights into their functional mechanisms, thereby inspiring a new generation of solutions against target pathogens.

## Materials and methods

### Materials

*S. typhimurium* ATCC 14028 and a human gastric cancer cell line were sourced from the Iranian Research Organization for Science and Technology (IROST, Tehran, Iran) and the Pasteur Institute of Iran (Tehran, Iran), respectively, while *Komagataeibacter xylinus* BPR 2001 was obtained from the culture collection of the Faculty of Veterinary Medicine at Urmia University. The materials used in this study included diethyl ether (≥ 99%, Scharlau, Barcelona, Spain), absolute ethanol (≥ 99.8%) and methanol (≥ 99.8%) (Zist Faravardeh Sepahan, Esfahan, Iran), xylose-lysine-desoxycholate (XLD) agar (Ibresco, Alborz, Iran), plate count agar (PCA) (Quelab, Montreal, Canada), trypticase soy broth/agar (TSB/TSA) (Ibresco Co., Karaj, Iran), and 2-thiobarbituric acid (TBA) (Quelab, Montreal, Canada). Gluten was obtained from Faravari Fructose NAB Company (Tehran, Iran), beeswax from BehdashtGhostar (Urmia, Iran), and molasses from the Miandoab Sugar Factory (Miandoab, Iran). PTFE filters (0.22 μm) were purchased from Millipore. (Burlington, USA). Additional reagents, including acetic acid (≥ 99%), citric acid (≥ 99.5%), glucose (≥ 99.5%), yeast extract, disodium hydrogen phosphate (≥ 99%), ammonium sulfate (≥ 99%), dimethyl sulfoxide (DMSO,

≥ 99.9%), peptone water and carboxymethyl cellulose (CMC; degree of substitution 0.9) were acquired from Merck (Darmstadt, Germany). DPPH (≥ 90%), MTT (≥ 98%), and other necessary materials were provided by Sigma-Aldrich (Madrid, Spain).

### Synthesis of BNC

According to the method described by Ghorbani et al. ^23^, BNC was obtained from *K. xylinus* BPR 2001 under static culture conditions. The first step was the preparation of Hestrin–Schramm (HS) medium containing 0.5% (w/v) peptone water, 2% (w/v) glucose, 0.5% (w/v) yeast extract, 0.15% (w/v) citric acid, and 0.27% (w/v) disodium hydrogen phosphate. After adjusting pH to 5.5 and sterilizing the medium by autoclaving, the bacterial strain was inoculated and incubated at 30 °C for 72 h. The initial layer of cellulose was subsequently removed. A modified HS medium [0.63% (w/v) ammonium sulfate, 1.91% (w/v) gluten, 5.38% (w/v) molasses, 0.27% (w/v) disodium hydrogen phosphate, 1.38% (w/v) ethanol, and 0.15% (w/v) citric acid] was then prepared. After preparation, 40 mL of this modified medium was added to 400 mL of the initial medium and incubated at 30 °C for another 72 h. The BNC film was carefully peeled off and subsequently washed at room temperature (20–25 °C), first with distilled water, followed by 1 M sodium hydroxide until the film turned white. Acetic acid was applied to bring the pH closer to neutrality. BNC was suspended in distilled water at a neutral pH, autoclaved, and stored at 4 °C until use.

### Preparation of JPs

Initially, hydrophobic CDs (HCDs) were synthesized from beeswax^24^. Specifically, 5 g of beeswax was dissolved in 80 mL of acetic acid and stirred for one hour. Subsequently, the mixture was transferred to a hydrothermal reactor equipped with a Teflon liner, sealed, and subjected to a temperature of 200 °C for 10 h. To purify the HCDs, the resultant solution was added to 300 mL of boiling deionized water and boiled for 15 min. The solution was then cooled and filtered through Whatman No. 42 paper, yielding a light green filtrate. Diethyl ether was then added, and the mixture was stirred for one hour. The supernatant, pale-yellow diethyl ether phase containing the HCDs, was separated with a burette and left at 32 °C to allow the solvent to evaporate, resulting in HCD isolation. Subsequently, a 0.5% CMC solution was prepared in deionized water, and the HCDs were dissolved in 5% (v/v) DMSO. A 2:1 ratio of HCD and CMC were mixed and stirred at 650 rpm at room temperature for 48 h to facilitate the self-assembly of JPs. All related spectral, chemical, microscopic, antioxidant, and antimicrobial characteristics were comprehensively evaluated in prior analyses^24^.

### Cytotoxicity of JPs

3-(4, 5-dimethylthiazol-2-yl)-2, 5-diphenyltetrazolium bromide (MTT) was used to determine the cytotoxicity of JPs against the human gastric cancer cell line (AGS)^25^. The assay helped determine the appropriate JPs concentrations for safe inclusion in BNC films to be applied in meat products. Cell viability (initial 1 × 10^4^ cells) after 24 h exposure to JPs (0.75, 1, 2, 3, 4, and 5 mg/mL) was calculated relative to an untreated control group using Eq. (1):

1 $$\:{\mathrm{Cell}}\:{\mathrm{viability}}\left( \% \right) = \frac{{{\mathrm{mean}}\:{\mathrm{ODtreatment}}}}{{{\mathrm{mean}}\:{\mathrm{ODcontrol}}}} \times 100$$

### BNC-JPs films preparation

**Fig. 1:**
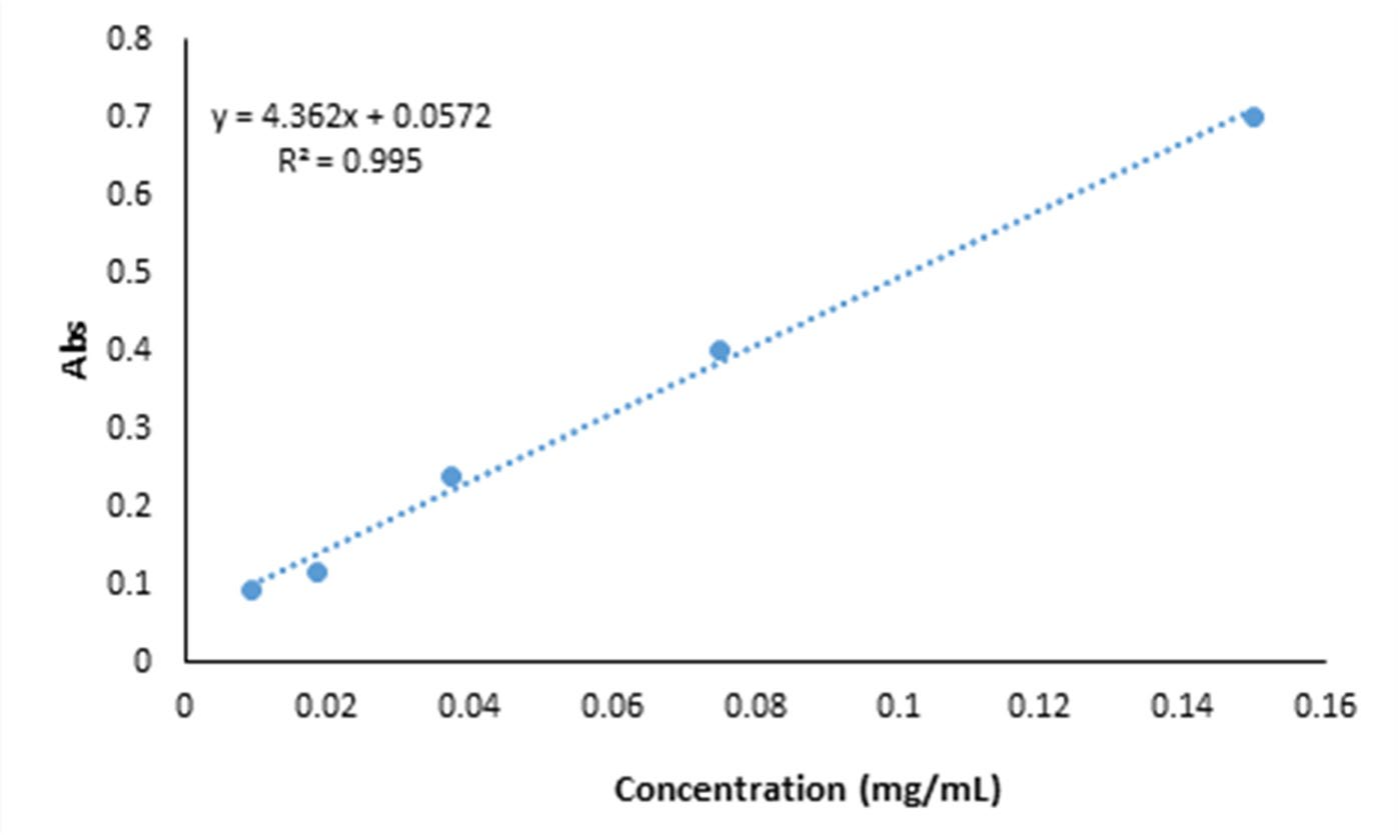
Standard adsorption calibration curve of Janus nanoparticles (JPs) in bacterial nanocellulose (BNC) films.

An *ex-situ* approach was used to prepare BNC-JPs films. For this purpose, BNC films with a thickness of 3 mm were placed between two glass plates to promote the extraction of approximately 70% of their water, as quantified by pre- and post-dewatering measurements. Subsequently, the BNC films were cut to a side length of 4.5 cm (diagonal ∼ 6.36 cm)^26^. The BNC pieces were then soaked in JPs solutions at concentrations of 0.01%, 0.02%, and 0.03% for 24 h at room temperature. BNC-JP films were tested at the same loading levels of 0.01%, 0.02%, and 0.03% owing to their safety and antibacterial considerations. The loading capacities were 0.07, 0.18, and 0.24 mg/cm³ for the BNC film. The JPs loading was calculated by monitoring the absorbance of the solution at 280 nm before and after immersion using a UV-VIS spectrophotometer (TG80 plus, China)^23,27^. Using a pre-established JPs absorption calibration curve and its corresponding equation, the precise amount of JPs incorporated into the BNC films was calculated (Fig. 1).

### Fourier transform infrared (FTIR) spectroscopy

FTIR spectroscopy (Nexus-670; Thermo Nicolet, USA) in attenuated total reflection mode was used to characterize surface functional groups and the chemical composition of BNC and BNC-JPs films in the wavenumber range of 650–4000 cm⁻¹.

### Field emission scanning electron microscopy (FESEM)

BNC films were oven-dried at 40 °C to preserve their structural integrity before imaging. The effect of JPs on the morphology and structural properties of the BNC films was examined using FESEM (SIGMA VP-300; Zeiss, Germany). The acceleration voltages.

The working distance for each image was 20 kV and 1–5 mm, respectively. Microstructural images showing the surface and the cross-section of the films were recorded at 20kx magnification.

### Mechanical properties

The mechanical characteristics, including tensile strength (TS), elastic modulus (EM), and elongation (E), of BNC and BNC-JPs films were evaluated using a texture analyzer (TA; XT-Plus, England).

### Antimicrobial performance

#### Minimum inhibitory and minimum bactericidal concentrations (MIC and MBC)

The MIC and MBC of the JPs were determined using the microdilution technique^28^. Initially, 160 µL of TSB was dispensed into each well of a 96-well microplate. Subsequently, 20 µL of the JPs dilutions were added to achieve final concentrations of 0.96, 0.48, 0.24, 0.12, 0.06, 0.03, 0.015, and 0.007 mg/mL. Thereafter, 20 µL of the bacterial suspension (10⁶ CFU/mL) was introduced into each well and incubated at 37 °C for 24 h. Bacterial growth or inhibition was evaluated by assessing turbidity or clarity. The minimum inhibitory concentration (MIC) was defined as the lowest concentration that inhibited visible bacterial growth. For MBC determination, 100 µL from the MIC well and two wells containing higher concentrations were inoculated onto culture medium. After 24 h of incubation at 37 °C, colony counts were used to establish the MBC as the minimum concentration that reduced the bacterial count by 99.99%.

#### Agar disc diffusion

The agar disc diffusion method was used to study the antimicrobial activity of BNC-JPs films against *S*. Typhimurium. A bacterial suspension of 10⁸ CFU/mL was spread evenly on TSB plates using a sterile cotton swab. Eight-mm-diameter discs prepared from BNC-JPs films were placed in triplicate on the inoculated plates in a regular pattern. The zone of inhibition (ZOI) was measured after 24 h of incubation at 37 °C using a digital caliper^29^.

### Antioxidant properties

The radical scavenging activity of the films was determined using the DPPH (2,2-diphenyl-1-picrylhydrazyl) assay^5^. The film containing JPs (0.01%, 0.02%, and 0.03%) (100 mg) was mixed in 2 mL of a 1:1 methanol/water solution and incubated for 3 h at room temperature. After extraction, 2 mL of the solution was mixed with 0.2 mL of 1mM methanolic DPPH solution and maintained in a dark place at room temperature for 30 min. The absorbance of all samples was measured at 517 nm. The percentage of radical scavenging (%RS) was calculated using Eq. (2):

2 $$\:\% RS = \frac{{{\mathrm{A}}_{{{\mathrm{blank}}}} - {\mathrm{A}}_{{{\mathrm{sample}}}} }}{{{\mathrm{A}}_{{{\mathrm{blank}}}} }} \times 100$$

where A _blank_ and A _sample_ represent the absorption rate of the DPPH solution and the DPPH solution containing the BNC-JPs extract, respectively.

### Release pattern

To study the release of JPs from BNC films, food simulants with varying ethanol concentrations (10%, 50%, and 95%) were prepared to represent aqueous solutions, oil-in-water emulsions, and fatty food components, respectively. The films were cut into squares (1.5 × 1.5 cm) and placed in conical centrifuge tubes containing 20 mL of each simulant solution. The samples were gently shaken at 100 rpm for 48 h, at ambient temperature. The JPs release was measured at definite time intervals (0, 4, 8, 12, 24, and 48 h) using a spectrophotometer at 280 nm^23,30^. The percentage of JPs release was calculated using the Eq. (3):

3 $$\:JPs\:release\left( \% \right) = \frac{{{\mathrm{M}}_{{\mathrm{t}}} }}{{\mathrm{M}}} \times 100$$

M_t_ represents the amount of JPs released from the film at time t (min), and M represents the initial concentration of JPs in the film.

### Application of BNC-JPs films on chicken breast meat

#### Antimicrobial efficacy of BNC-JPs films against *S. typhimurium*

Fresh chicken breast (24 h post-mortem) was obtained from a certified supplier (Behkam Kabir, Urmia, Iran) and transferred under sterile refrigerated conditions to the laboratory. BNC films were cut into 4.5 × 4.5 cm squares, sterilized with UV light for 2 min, and soaked in JPs solutions of concentrations of 0.01%, 0.02%, and 0.03%. *S.* Typhimurium (10⁷ CFU/mL) was prepared, and each piece of chicken breast (weight 10 g) was dipped in a mixture containing 20 mL of the bacterial suspension and 180 mL of peptone water for 2 min. Each chicken piece inoculated in this manner was then completely wrapped with two layers of film, placed separately into sterile polyethylene trays, and stored at 7 °C for up to 16 days. Surface culture method on XLD agar plates was used for microbial analysis on days 0, 4, 8, 12, and 16. Samples were divided into five groups: control (no film), pure BNC, BNC-JPs_0.01%_ (loading: 0.07 mg/cm³), BNC-JPs_0.02%_ (loading: 0.18 mg/cm³), and BNC-JPs_0.03%_ (loading: 0.24 mg/cm³).

#### Shelf life assessment

To assess the shelf life of chicken breast, un-inoculated samples, both coated with film and untreated, were prepared as previously described. Comprehensive assessments, including microbiological, chemical, sensory, and color analyses, were conducted at 7 °C on days 0, 4, 8, 12, and 16.

##### Microbial analysis

For microbial evaluation, 10 g of each sample was aseptically mixed with 90 mL sterile 0.1% peptone water. The mixture was homogenized using a stomacher (Seward Medical Ltd., London, UK) at 260 rpm for 3 min. Serial dilutions were performed, and total mesophilic counts (TMC) were determined using the pour plate method after 48 h of incubation at 37 °C^1^.

##### Chemical analysis

The effects of BNC-JP films on meat quality were assessed by measuring pH, total volatile basic nitrogen (TVB-N), and thiobarbituric acid reactive substances (TBARS). The pH of the homogenized samples was recorded using a calibrated pH meter (Metrohm, Herisau, Switzerland). TVB-N levels were measured using the Kjeldahl procedure^1^, and TBARS values, representing lipid oxidation, were determined from malondialdehyde content measured at 532 nm.

##### Sensory and color analysis

The color attributes lightness (L*), red/green (a*), and yellow/blue (b*) were measured using a Lovibond^®^ LC 100 Spectrocolorimeter (Tintometer^®^ Group, Lovibond House, UK). For sensory evaluation, raw meat samples were randomly coded and assessed by a semi-trained panel of 10 members (three males and seven females; 24–34 years old). Participants rated odor, color, and overall acceptability using a 9-point hedonic scale (9 = like extremely; 1 = dislike extremely). Although the researchers did not obtain formal ethics committee approval or written consent forms, they adhered to ethical practice guidelines regarding the privacy and rights of the participants. The authors confirm that all participants provided informed consent prior to participation.

### Statistical analysis

All experiments were conducted in triplicate. Data were analyzed using analysis of variance (ANOVA) followed by Duncan’s multiple range test with GraphPad Prism software version 8.4.1 (San Diego, CA, USA). Statistical significance was set at 5% (*P* < 0.05) using Tukey’s post hoc test.

## Results and discussion

### JPs characterization

According to Razavi et al.^24^, the synthesized JPs have a spherical CMC core densely decorated with ultrasmall HCDs and a rough surface, as observed by FESEM. The average diameter of the JPs was approximately 3.99 nm, with a narrow size distribution, and 99% of the particles had diameters below 10.8 nm. FTIR of the JPs showed characteristic O-H, C-H, and C = O/C-O vibrations, along with evidence of interaction between HCDs and the CMC core, while XRD demonstrated a graphitic feature in the HCDs (2θ: 22.15°), and JPs pattern included peaks corresponding to crystalline phases of CMC.

### Cytotoxicity of JPs

**Fig. 2:**
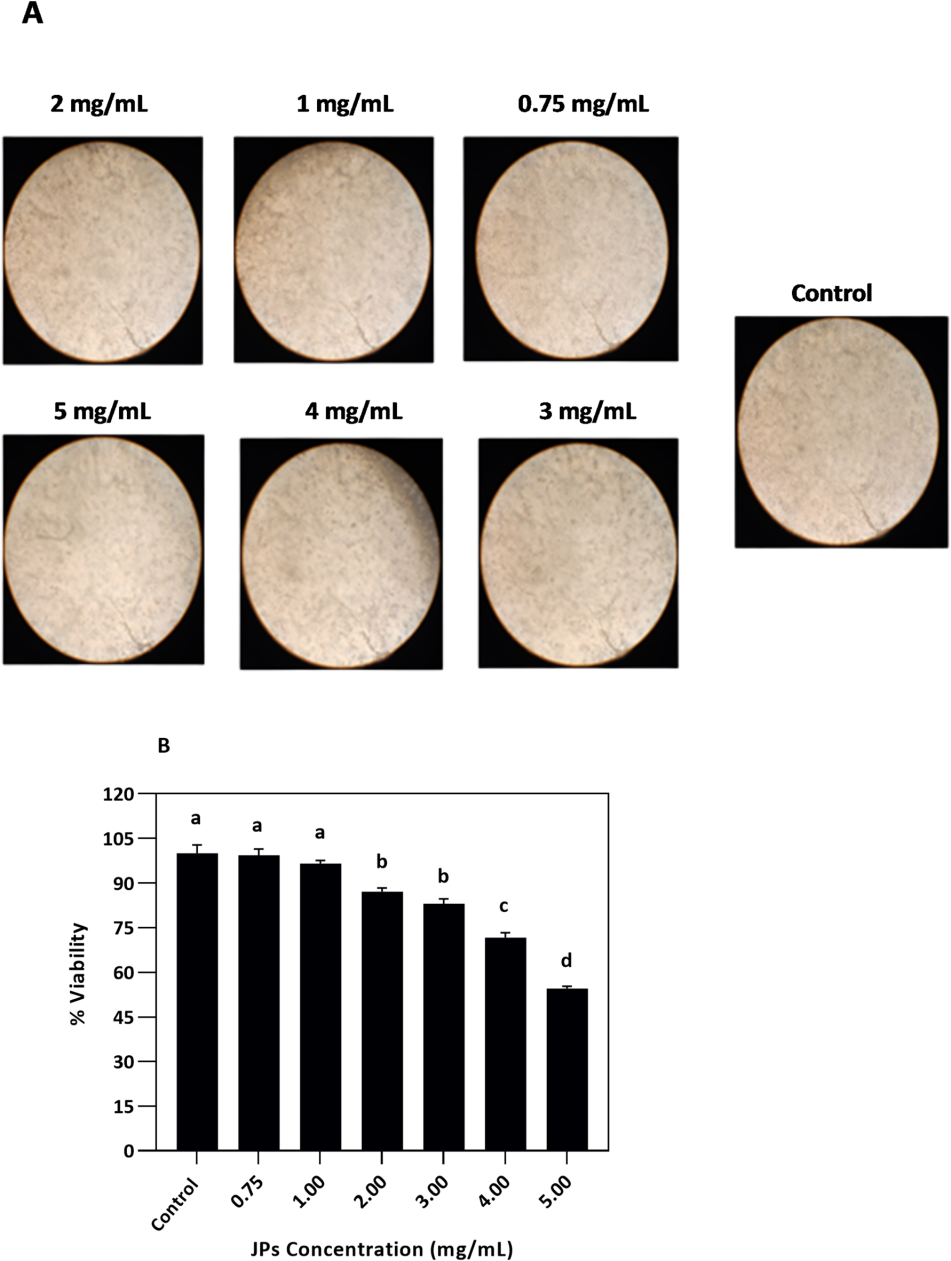
Microphotographs of human gastric cancer cells (AGS) (**A**) and percentage viability (%) (**B**) after treatment with varying concentrations of Janus nanoparticles (JPs) for 24 h.

It is crucial to conduct a thorough analysis of the potential toxicological effects of novel materials to establish their safe application. In the present study, we examined whether JPs at different concentrations exhibited cytotoxic activity in AGS cells using the MTT assay. As indicated in Figs. 2 and 5 mg/mL JPs displayed significant toxicity compared to the control. Microscopic observation at 100× magnification revealed morphological changes in AGS cells (Fig. 2) treated with JPs. JPs at a concentration of 5 mg/mL caused evident morphological changes, including weakened and distorted cell walls, increased cell opacity, and eventual cell death. Although CMC is a biodegradable polymer with low toxicity, the presence of surface functional groups on CMC and HCDs may affect their interactions with biological systems. The JPs possess a typical amphiphilic structure that enables them to interact specifically with biological environments and cell membranes, which may lead to distinct toxicity pathways. The hydrophobic component can embed itself into the lipid bilayer, potentially disrupting membrane integrity. Such disruptions may lead to increased membrane permeability, altered ionic balance, and even cell lysis.

Moreover, HCDs with surface defects or quantum confinement characteristics have the potential to generate reactive oxygen species (ROS), which may cause oxidative damage to DNA, proteins, and lipids, and consequently induce apoptosis^31^. Likewise, Razavi et al.^24^ examined the impact of HCDs synthesized from beeswax and JPs fabricated from HCDs and CMC on the L929 mouse fibroblast cell line. HCDs were toxic at lower concentrations (1.5 mg/mL), whereas JPs were cytotoxic only at higher concentrations (5 mg/mL). The authors suggested that greater cell viability with JPs could be due to the hydrophilic constituents in JPs, which appeared less toxic than HCDs alone. Similarly, Zhang et al.^32^ reported a notable reduction in HepG2 cell viability when treated with 0.2–0.25 mg/mL tea saponin-based HCDs with high cytotoxicity. Owing to their lipophilicity, nanoscale hydrophobic particles can easily penetrate cell membranes, potentially damaging membrane proteins and altering their functions. The biocompatibility and toxicity of CDs depend on various factors, including their size, surface properties, and chemical structure^31^.

### FTIR analysis of films

**Fig. 3:**
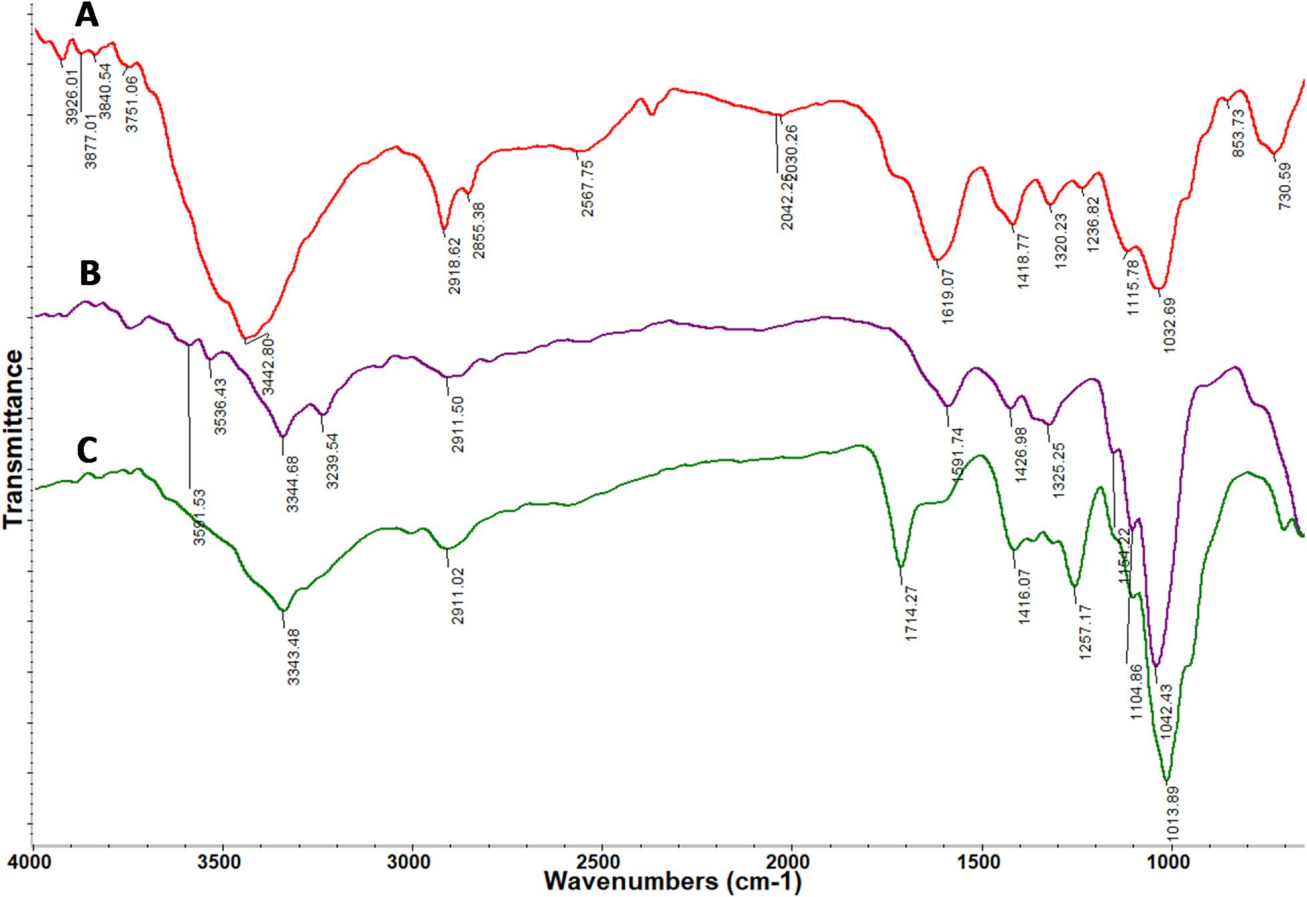
FTIR spectra of Janus nanoparticles (JPs, A), bacterial nanocellulose (BNC, B), and BNC- JPs_0.03%_ (C) in the range of 650–4000 cm^− 1^.

To identify the functional groups in the synthesized JPs, BNC, and BNC-JPs films, FTIR spectra were recorded over the range of 650–4000 cm⁻¹ (Fig. 3). In JP’s FTIR spectrum (Fig. 3A), strong absorption bands were observed at 3442, 2918, 2855, 1619, and 1418 cm⁻¹, corresponding to O-H stretching, C-H stretching (methyl and methylene groups), C = O bending, and C-O bending, respectively. The C-C bond characteristic of pyranoid rings appeared between 1000 and 1200 cm⁻¹ ^24^. Conversely, the BNC (Fig. 3B) spectrum exhibited characteristic peaks at 3344 cm⁻¹, 1591 cm⁻¹, 1325 cm⁻¹, and 1426 cm⁻¹, representing O-H, C = O stretching, and COOH bending, respectively. Furthermore, the strong and broad bands in the 1000–1200 cm⁻¹ region confirmed the presence of glycosidic ether linkages and pyranoid rings in the synthesized BNC^33^. In spectrum Fig. 3C, corresponding to the BNC-JPs film, there are distinctive absorption peaks for O-H (3343 cm⁻¹), COOH/O-H (1416 cm⁻¹), C = O (1714 cm⁻¹), and vibrations corresponding to glycosidic ether linkages and pyranoid rings (1013 cm⁻¹). Notable differences between unmodified BNC and JPs-modified BNC include the disappearance of the hydroxyl stretching at 3239 cm⁻¹, a C = O vibration shift from 1619 cm⁻¹ to 1714 cm⁻¹, and a COOH/O-H bending shift from 1426 cm⁻¹ to 1416 cm⁻¹, which suggest that Janus nanostructures were successfully integrated, likely through interactions involving the highlighted functional groups^34^.

### FESEM specifications

**Fig. 4:**
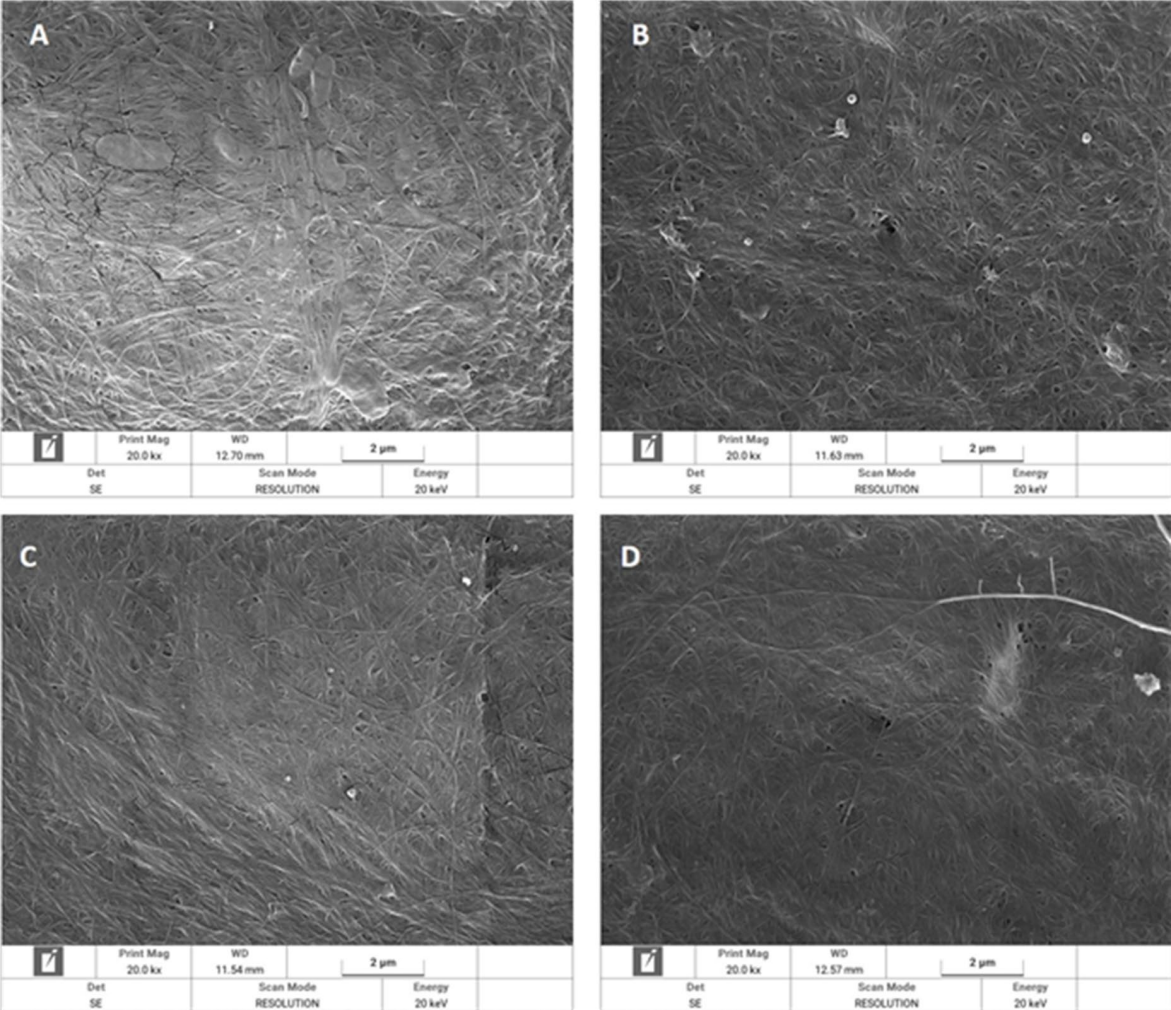
FESEM images of bacterial nanocellulose (BNC) film (**A**) and BNC with Janus nanoparticles (JPs) of 0.01% (**B**), 0.02% (**C**), and 0.03% (**D**) concentrations.

The porous structure and hydroxyl-rich surface of BNC make it highly receptive to guest molecules. However, the incorporation of external substances can affect the uniformity, cohesion, structural integrity, and density of the BNC matrix. Previous studies have shown that BNC morphology is influenced by the concentration, molecular structure, and inherent properties of the incorporated materials^6^. The FESEM images (Fig. 4A) reveal a BNC nanofibrillar architecture, confirming the dense and porous network. As expected, the *ex-situ* incorporation of JPs did not notably alter this structure; instead, the fibrillar gaps and irregular pores were filled (Fig. 4B, C, and D). The uniform interfibrillar spacing in the treated samples was attributed to the low concentration of JPs used.

### Mechanical properties

One of the most important benefits of BNC is its ability to form films (membranes) with a uniform thickness. These membranes are mechanically robust and flexible and can be used directly in packaging after drying^9^. The mechanical behavior of BNC is affected by various factors, including the type and quantity of incorporated nanomaterials, incorporation method, and degree of dispersion of the additive in the BNC matrix. Khan et al.^35^ reported that incorporating polyvinyl alcohol (PVA) and silver nanoparticles (AgNPs) into BNC improved mechanical properties. PVA enhanced the tensile strength and Young’s modulus, whereas the elongation at break decreased slightly. Moreover, the tensile strength initially improved with higher concentrations of AgNPs, but it began to decrease beyond a certain level. In another study, loading BNC with CDs synthesized from postbiotics at a concentration of 22.5 mg/cm^3^ considerably increased the elongation at break but simultaneously decreased the ultimate tensile strength and Young’s modulus^6^. However, Mirtalebi et al.^36^ found that incorporating MgO nanomaterials into BNC via an in-situ method did not significantly alter the elongation at break, possibly because of the lower loading capacity. In contrast, our incorporation of JPs into BNC modified its mechanical properties, reducing both tensile strength and elongation at break. The elastic modulus showed a non-monotonic response to JPs concentration; it increased at 0.01–0.02% loading and decreased at 0.03% loading compared with neat BNC (Table 1). The loss of tensile strength and elongation at break with the addition of JPs arises from the biphasic and partially hydrophobic nature of JPs. The hydrophilic CMC fragment intermeshed with BNC, and the HCD regions might have formed minute cracks and holes within the highly hydrophilic cellulose region^37^. These holes act as stress concentrates, causing microcracks and hence premature failure. Thus, there would be a loss of extensibility as a result of tensile strength and elongation at break^38^. The elastic modulus followed a nonlinear path. It initially increased slightly at low loading concentrations (0.01–0.02%) and decreased at 0.03%. This can indicate that very low concentrations of nanoparticles might be suppressing the mobility of the polymer chain sufficiently to make it stiffer, and at the highest concentration, gaps between interfaces and nanoparticle aggregation might be causing it to be less stiff^39^.

**Table 1:** The mechanical properties of bacterial nanocellulose (BNC) with and without Janus nanoparticles.

	Elongation at Break (%)	Ultimate Tensile Strength (MPa)	Elastic Modulus (MPa)
BNC	43.7 ± 1.6^a^	168.8 ± 1.5^a^	331.9 ± 0.7^c^
BNC-JPs _0.01%_	42.7 ± 3.5^a^	82.4 ± 2.3^c^	353.9 ± 1.4^b^
BNC-JPs _0.02%_	42.1 ± 2.9^a^	125.2 ± 3.1^b^	421.3 ± 3.0^a^
BNC-JPs _0.03%_	40.0 ± 0.6^a^	62.6 ± 3.5^d^	183.3 ± 1.8^d^

Within each column, values with different letters are significantly different (*P* < 0.05).

### Antibacterial activity

**Fig. 5:**
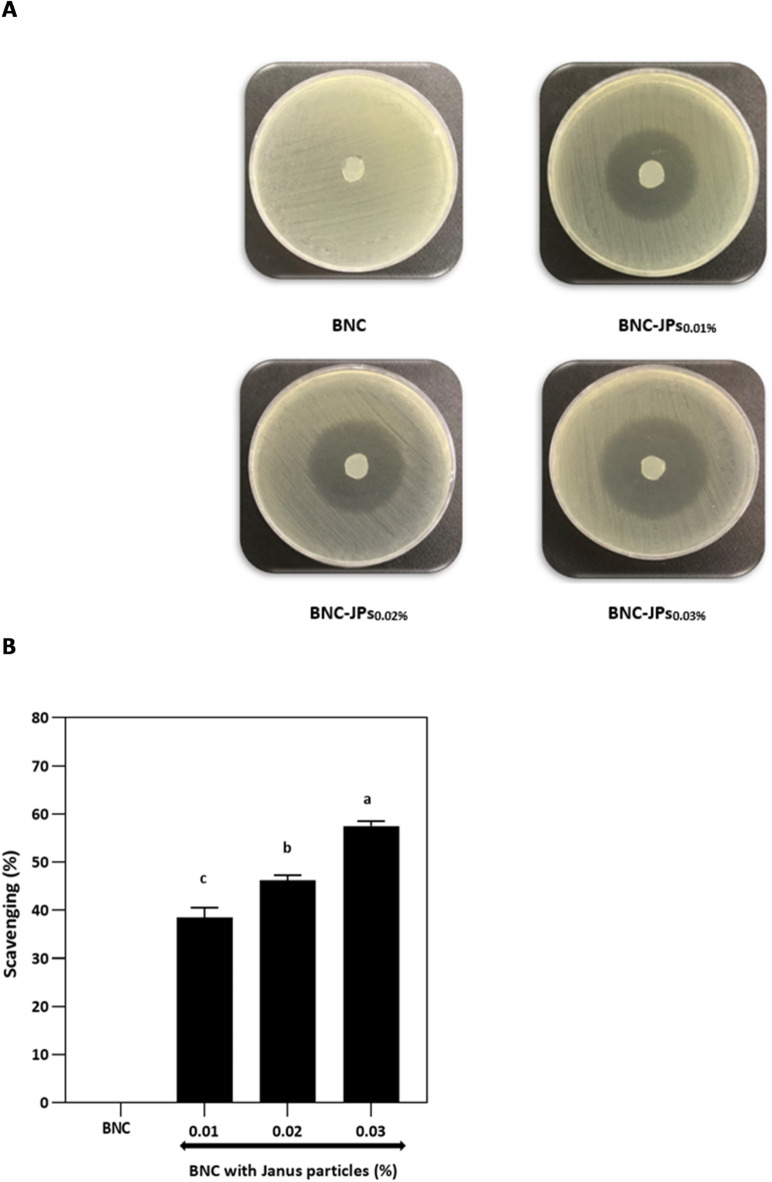
(**A**) Bacterial nanocellulose (BNC) film antimicrobial activity with and without JPs according to agar disc diffusion method as a diameter of zone of inhibition (mm). (**B**) Radical scavenging activity of bacterial nanocellulose (BNC) and BNC films with Janus nanoparticles (JPs) (BNC-JPs_0.01%_, BNC-JPs_0.02%_, and BNC-JPs_0.03%_) using DPPH assay.

The microdilution method was used to determine the MIC and MBC values for *S*. Typhimurium, both of which were 0.03 mg/mL for JPs. The activity of the BNC-JPs films against *S*. Typhimurium was also evaluated by the disc diffusion test, which showed concentration-dependent antibacterial activity. Specifically, ZOIs for BNC films containing 0.01%, 0.02%, and 0.03% JPs were 17.1 ± 0.5 mm, 20.7 ± 1.3 mm, and 27.5 ± 1.5 mm, respectively (Fig. 5A). The anisotropic structure of the JPs provides dual functionality: the hydrophilic CMC side facilitates dispersion and biocompatibility with living systems, while the HCDs side disrupts microbial membranes. This asymmetrical design facilitates attachment to bacterial surfaces and membrane destabilization, thereby promoting antimicrobial activity while potentially reducing resistance development. The outer membranes of Gram-negative bacteria, which are hydrophobic, are likely to be resistant to hydrophilic antimicrobials^40^. Conversely, hydrophobic antimicrobials can more readily permeate membranes by inserting into the lipid bilayer^41^. Owing to their Janus-like nature, JPs can penetrate both Gram-positive and Gram-negative bacteria. Their small size and high surface-to-volume ratio enable them to destroy bacteria through oxidative stress, alterations in membrane permeability, and damage to DNA, RNA, and proteins^42^.

Yuan et al.^43^ reported the creation of a Janus biopolymer composite coating using modified quaternized chitosan and aldehyde carboxycellulose nanofibers, which demonstrated antibacterial activity against *E. coli* and *S. aureus*. The observed bacteriostatic zone exceeded 10 mm, indicating effectiveness against both Gram-negative and Gram-positive bacterial strains. Other studies have developed lotus-like Janus films (L-SPGBC) from soybean polysaccharides (SPS), SPS-stabilized silver nanoparticles (AgNPs), and gelatin for the hydrophilic surface, with carnauba wax providing the hydrophobic surface. While SPS films were not antibacterial, the L-SPGBC films successfully inhibited over 95% of *E. coli* and *S. aureus* due to the release of bactericidal Ag⁺ ions, which created clear inhibition zones^44^. These results confirm the strong and broad antibacterial efficacy of JPs.

### Antioxidant activity

The DPPH assay was used to evaluate the antioxidant activity of BNC-JPs films. Figure 5B shows that pure BNC exhibited no radical scavenging activity, indicating that it has no inherent antioxidant activity^5^. However, the addition of JPs significantly increased BNC’s radical scavenging ability of BNC, with the effect being proportional to the JP concentration. Several factors contribute to the observed antioxidant activity. The ability to donate electrons, combined with the highly reactive surface of HCDs, renders them effective radical scavengers. CMC acts as both a stabilizer and thickening agent, thereby broadening the protective effects of JPs. It stabilizes the solution while simultaneously boosting its free radical scavenging capacity^45^. FTIR spectroscopy also revealed that O-H, COOH, and C-O functional groups on the surface of JPs are responsible for their radical-scavenging ability. Beeswax, the HCD precursor, consists of proteins, minerals, and polyphenols that provide additional antioxidant benefits^46^. This synergistic combination allows for continuous neutralization of ROS. In the present study, potent antioxidant effects were achieved using very low concentrations (0.01–0.03% w/v) of biphasic JPs. The biphasic nature of JPs enhances their ability to exhibit antioxidant activity. This superior performance is attributed to the biphasic structure of the nanoparticles, which provides a highly reactive surface area and their effective stabilization within the BNC network.

### Release properties

**Fig. 6:**
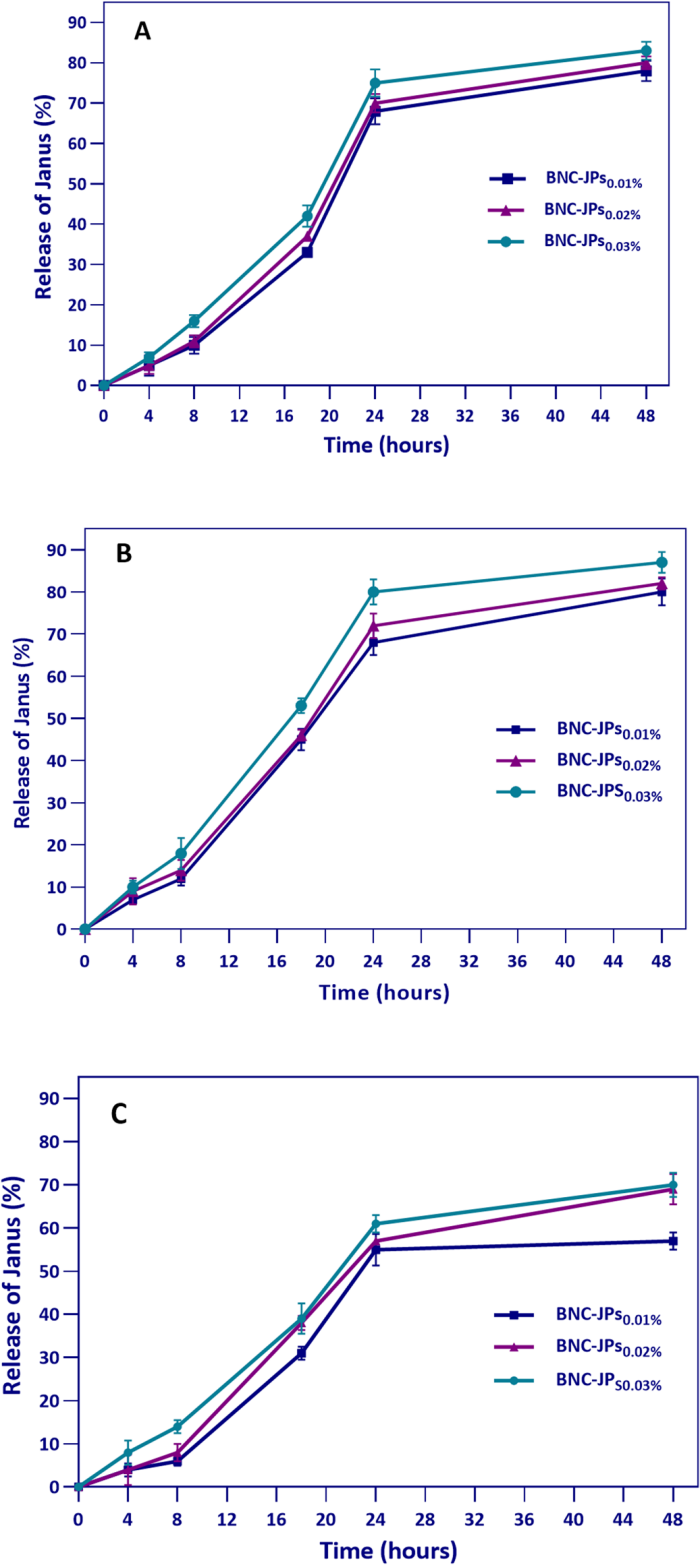
Release rate of the Janus nanoparticles (JPs) from the bacterial nanocellulose (BNC) film (BNC-JPs_0.01%_, BNC-JPs_0.02%_, and BNC-JPs_0.03%_) in various food simulant solutions [10% (**A**), 50% (**B**) and 95% ethanol (**C**)] after exposure for 48 h.

Figure 6 shows the release profiles of JPs from BNC films at concentrations of 0.01%, 0.02%, and 0.03% in various food simulants. The unique structural properties of JPs enable their impressive release across all tested simulants. Due to their amphiphilic nature, rapid release occurred in 50% ethanol (an oil-in-water emulsion) (Fig. 6). Moreover, increasing JPs concentrations correlated with faster release rates, confirming concentration-dependent release kinetics. The rapid and extensive release in ethanol-based simulants likely improves antioxidant effectiveness by enhancing HCDs availability for free radical neutralization. In contrast, in water-based simulants, the release was slower, suggesting a more sustained but delayed antioxidant effect, indicating medium-dependent variations in JP performance. The migration of active substances from a biodegradable film into food simulants depends on multiple factors, such as the simulant’s chemical properties, the film’s solubility, and the compound-film binding affinity^47^. Intermolecular interactions, water retention capacity, pore size, and nanoparticle distribution uniformity within the BNC network control nanoparticle release^48^. Ghorbani et al.^23^ investigated the release kinetics of CDs synthesized from *Saccharomyces cerevisiae* postbiotics in BNC films into food simulants. Their results demonstrated that increased CDs concentrations accelerated release rates, whereas higher alcohol content in simulants had an inhibitory effect on the release process.

### Application of BC-JPs films on chicken breast meat

#### Antibacterial effectiveness against *S. typhimurium*

**Fig. 7:**
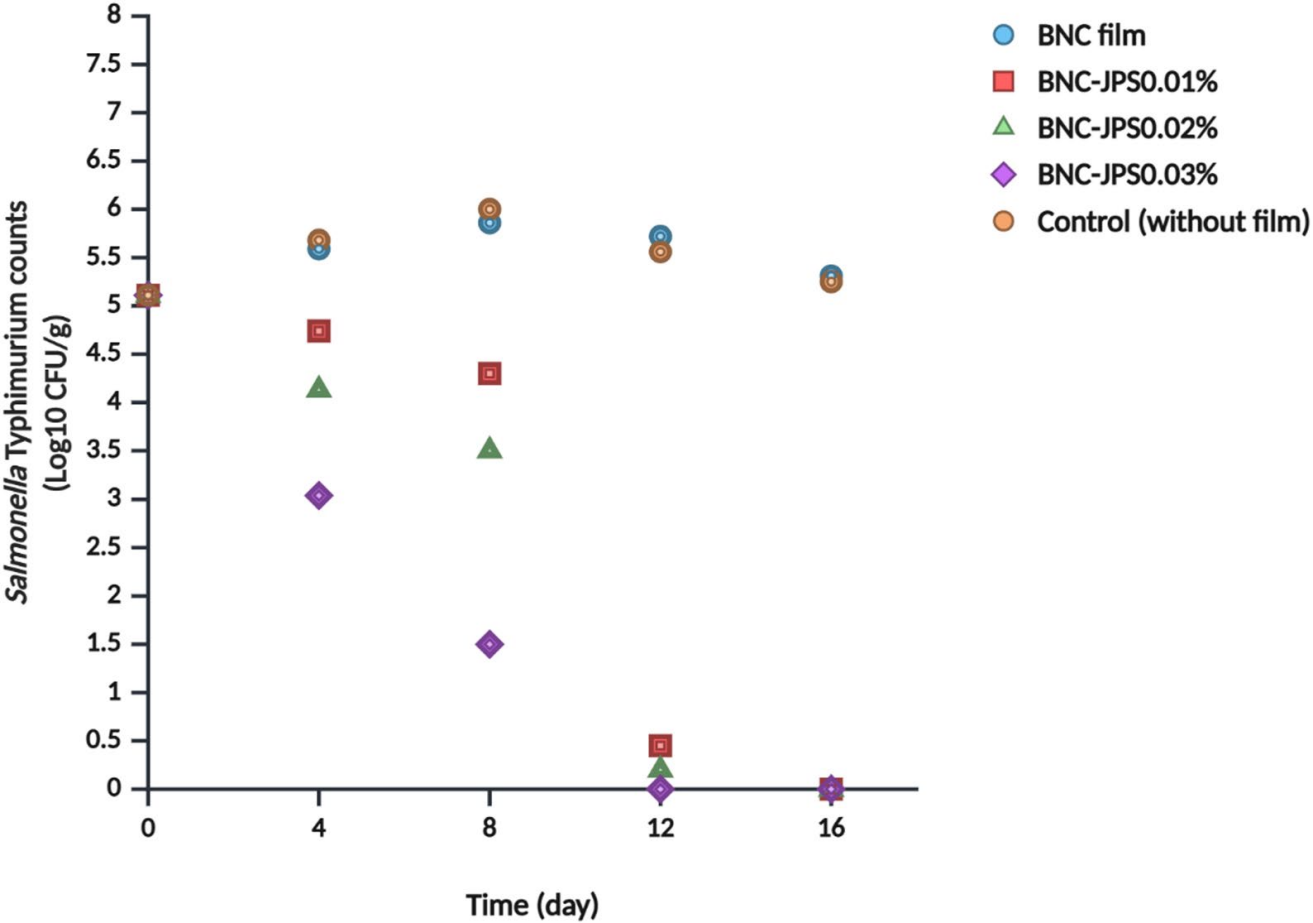
Efficacy of bacterial nanocellulose (BNC) and BNC with Janus nanoparticles (BNC-JPs) on *Salmonella* Thyphimurium counts (Log_10_ CFU/g) in chicken beast during 7 °C storage.

Figure 7 shows the antimicrobial efficacy of these films against *S*. Typhimurium in chicken breast. Over 16 days, bacterial counts in control samples increased slightly from 5.1 log₁₀ CFU/g to 5.2 log₁₀ CFU/g. This minimal population change in the control sample may be due to reduced competitiveness resulting from elevated counts of psychrotrophic and mesophilic bacteria, as well as increased pH in the chicken breast meat^49^. Similarly, samples treated with the pure BNC film showed an increase of 5.3 log₁₀ CFU/g. In comparison, BNC-JP-packaged samples demonstrated remarkable bacterial reduction after 12 days, from 5.1 log₁₀ CFU/g (day 0) to 0.45, 0.3, and undetectable levels for 0.01%, 0.02%, and 0.03% JPs, respectively. By day 16, all JP-containing samples achieved complete bacterial inhibition (undetectable status). These findings reflect the strong antibacterial efficacy of BNC-JPs films against *S*. Typhimurium at low temperatures, with higher JPs concentrations yielding greater inhibition. These results are consistent with those of other nanoparticle studies. For example, Riahi et al.^10^ reported that cellulose nanofiber/pullulan films incorporating Zn-doped CDs from avocado considerably inhibited *L. monocytogenes* and *E. coli* on chicken and tofu, whereas films without nanoparticles did not exhibit antibacterial activity. Similarly, Lin et al.^50^ reported that gelatin films containing eugenol nanoparticles reduced *S. aureus* in chicken by approximately 4 log₁₀ CFU/mL more than control samples.

#### Shelf life assessment

##### Microbial analysis

**Fig. 8:**
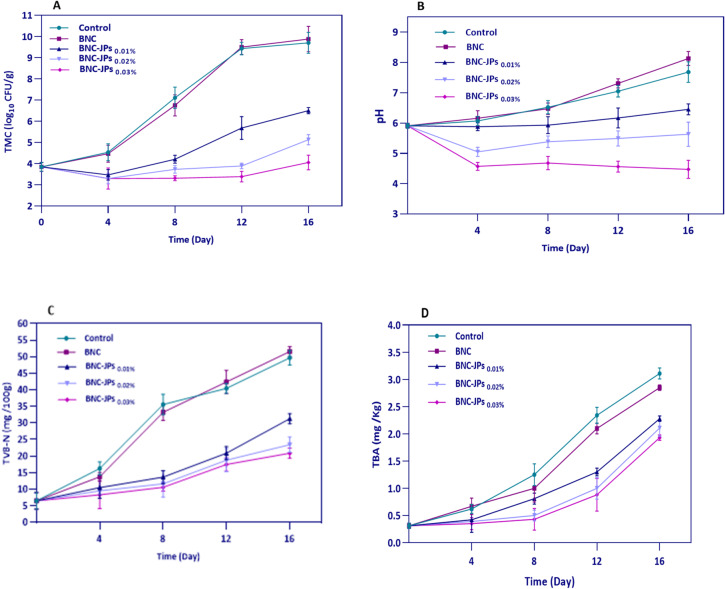
Total mesophilic bacteria count (**A**); pH (**B**), total volatile basic nitrogen (**C**), and thiobarbituric acid reactive substances (**D**) analysis of chicken breast packaged with bacterial nanocellulose (BNC) and BNC with Janus nanoparticles (BNC-JPs) during 7 °C storage.

Figure 8A shows the antimicrobial efficacy of BNC films with and without JPs in inhibiting mesophilic bacterial growth on chicken breast. TMC increased from 3.8 log₁₀ CFU/g on day 0 to 9.7 log₁₀ CFU/g (control) and 9.9 log₁₀ CFU/g (pure BNC) on day 16. In contrast, BNC-JPs-treated samples showed significantly suppressed bacterial growth, with TMCs of 6.5 log₁₀ CFU/g (0.01% JPs), 5.1 log₁₀ CFU/g (0.02% JPs), and 4.1 log₁₀ CFU/g (0.03% JPs) on day 16. These results represent reductions of 3.2, 4.6, and 5.6 log₁₀ CFU/g, respectively, compared to the control (*P* < 0.05). Notably, by day 4, all JP-treated samples exhibited a 1 log₁₀ CFU/g reduction from their initial TMC values, indicating a potential burst release of JPs. The antibacterial efficacy gradually diminished over time, likely due to progressive depletion of JPs from the films.

Razavi et al.^24^ also reported similar trends, where the use of JPs and HCD at 0.05% through direct addition maintained the TMC below 5 log₁₀ CFU/g in ground beef for 9 d, while the control samples exceeded 7 log₁₀ CFU/g. Ma et al.^44^ developed a double-layer Janus film comprising soybean polysaccharides, silver nanoparticles, gelatin, and wax on one side and carnauba wax on the opposite side that effectively prolonged the shelf life of poultry, swine, and grapes. Similarly, Sasikumar et al.^51^ demonstrated that PVA/gelatin films incorporating triphala-based CDs effectively inhibited bacterial growth in chicken by 3 log₁₀ CFU/mL compared with controls. Collectively, these studies highlight the bioactive packaging potential to delay spoilage, with effectiveness depending critically on active agent concentration, release kinetics, and compatibility with the packaging matrix.

##### pH value

Microbial proteolysis of meat produces nitrogenous alkaline substances, such as ammonia and amines, which are responsible for elevated pH levels. The typical pH value for fresh meat is generally below 6^2^. Figure 8B shows the pH changes in raw chicken breast samples under different packaging treatments. The initial pH was 5.9, indicating product freshness, whereas on day 16, the pH values for the BNC-JPs-treated groups at 0.01%, 0.02%, and 0.03% were 6.4, 5.6, and 4.5, respectively, which were much lower than those of the control (7.7) and pure BNC (8.1). The observed pH reduction is most likely due to the continuous leaching of JPs into the meat, along with their natural acidity, which opposes the pH increase typically associated with spoilage.

##### TVB-N

Volatile amines, such as ammonia, dimethylamine, and trimethylamine, are produced during the proteolytic breakdown of meat. Various studies have adopted different TVB-N thresholds for chicken: 15 mg/100g^52^, 25 mg/100g^53^, and 28 mg/100g^54^. As the TVB-N content in meat results from microbial activity, adding antimicrobial substances to packaging films or coatings can prevent spoilage. In the current study, TVB-N content in chicken samples stored at 7 °C increased gradually (Fig. 8C), with an average of approximately 6.5 mg/100 g on day 0. During the first 8 days, BNC-JPs-treated samples showed relatively lower TVB-N levels, indicating reduced microbial activity. This protective effect can be attributed to the rapid migration of JPs from the BNC film to meat surface. By day 16, control and BNC-only samples showed significant increases in TVB-N, reaching 49.68 mg/100 g and 51.55 mg/100 g, respectively. JPs-treated samples, however, demonstrated better preservation, with final TVB-N values of 31.31 mg/100 g, 23.45 mg/100 g, and 20.88 mg/100 g at 0.01%, 0.02%, and 0.03% levels, respectively. These findings align with previous reports on the impact of JPs-based films, such as a biodegradable cellulose/curcumin Janus film designed for real-time visual freshness tracking. Compared to commercial polyethylene films, this Janus film markedly decreased the accumulation of volatile alkaline nitrogen in stored tilapia fillets by 4.67 mg/100 g^55^. In another study, the Janus zein/chitosan film loaded with tannic acid and cinnamon essential oil Pickering emulsion slowed TVB-N accumulation, keeping values closer to the limit through 15 days, thus extending the pork shelf life by inhibiting microbial growth and microbial-induced protein degradation^21^.

##### TBARS value

Lipid and protein oxidation significantly affects the quality and shelf life of meat products. The TBARS value is commonly used to screen for secondary oxidative byproducts, such as aldehydes and polyunsaturated fatty acid breakdown products^2^. There is no universal TBRS threshold, as levels vary depending on species, diet, age, processing conditions, and test methods^56^. Zhou et al.^57^ suggested that TBARS values above 0.5 mg MDA/kg in chicken can produce perceivable off-flavors. In this study, all samples showed a steady increase in TBARS values during storage (Fig. 8D). The initial TBARS value was 0.3 mg MDA/kg. By day 8, samples containing JPs at 0.02% and 0.03% had TBARS values below 0.5 mg MDA/kg. Control samples reached 3.1 mg MDA/kg by day 16, while pure samples treated with unmodified BNC showed 2.8 mg MDA/kg. In contrast, BNC-JP films considerably reduced oxidative damage, with final TBARS values of 2.3, 2.1, and 1.9 mg MDA/kg for the 0.01%, 0.02%, and 0.03% treatments, respectively. These results highlight a strong antioxidant capacity of BNC-JPs films in preventing free radical-induced oxidation in a dose-dependent manner. In a previous study, incorporating JPs and HCDs at 0.01% and 0.05% into minced meat showed increased TBA during storage, but JPs_0.05%_ and HCD_0.05%_ had the lowest oxidation, with 9-day values of 0.64 and 0.7 mg MDA/kg, respectively, below the freshness threshold^24^.

##### Sensory and color analysis

**Fig. 9:**
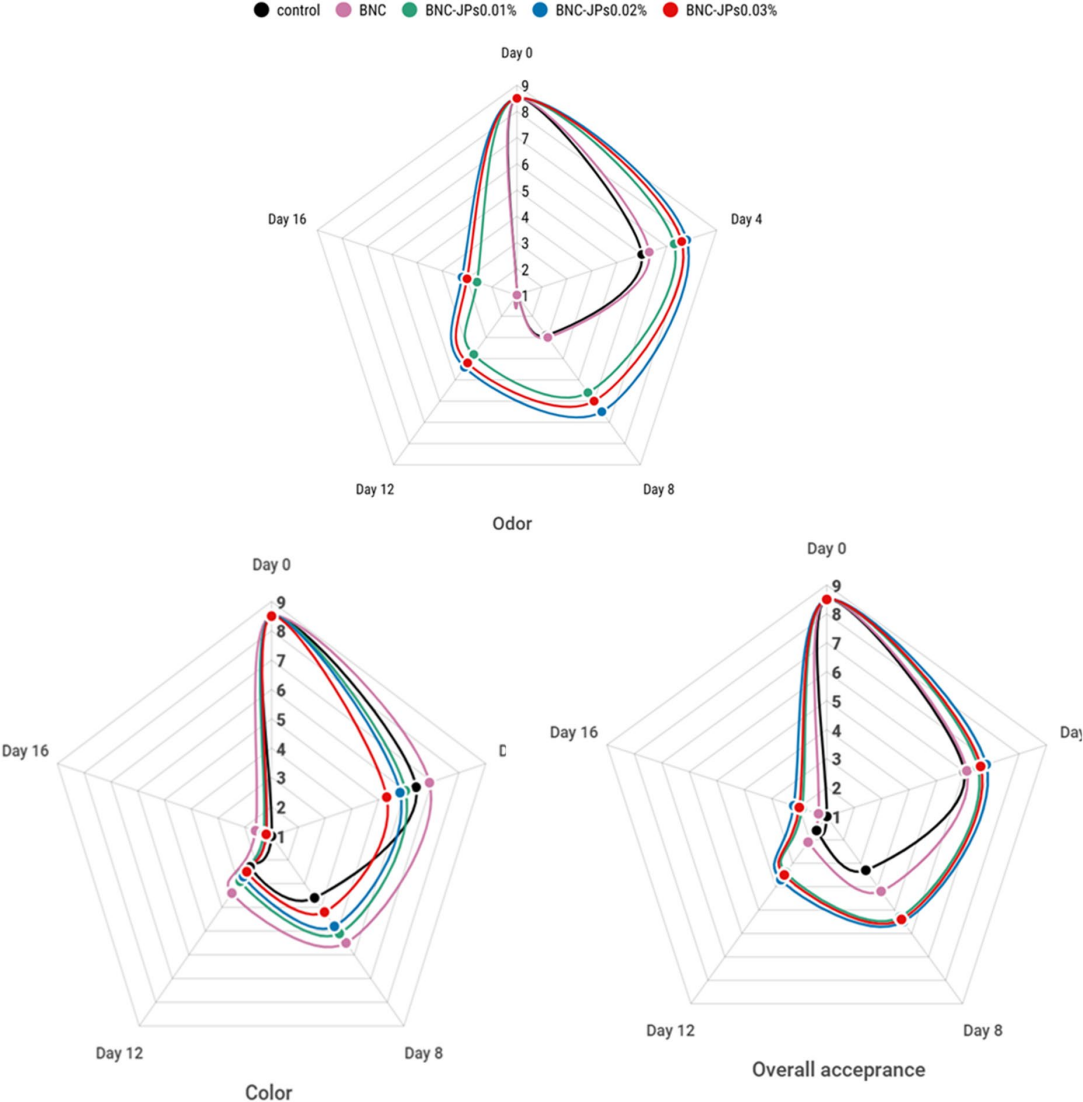
Sensory changes on different days in control and chicken breast treated by bacterial nanocellulose (BNC) and BNC with Janus nanoparticles (BNC-JPs) during 7 °C storage.

Sensory analyses of the control and treated chicken samples were conducted at four-day intervals over a 16-day storage period (Fig. 9). Initially, the assessors detected a strong vinegar-like aroma in the JPs-treated samples, which was slightly offensive. This aroma is highly likely due to the release of volatile components from the amphiphilic Janus structure, which can partition differently in the food matrix. In contrast, after extended storage, both untreated and control BNC samples developed stronger and more objectionable off-odors. Meanwhile, the vinegary aroma typical of the JPs-treated samples diminished and became more acceptable over time. Visually, JPs-treated chicken breasts tended to become lighter and whiter over time. Among all the tested samples, the one treated with the lowest concentration of JPs (0.01%) ranked highest in terms of visual appeal, odor acceptability, and overall sensory quality. These findings indicate the dual role of Janus particle-loaded BNC films: they not only inhibit spoilage-related odor development but also preserve acceptable visual and sensory properties. The effects of BNC-JPs films at various concentrations (0.01%, 0.02%, and 0.03%) and pure BNC on the color indices (L*, a*, and b*) of chicken meat were investigated during 16 days of refrigerated storage (Table 2). Throughout storage, the L* (lightness) value decreased for all samples at varying rates. The greatest reduction in L* was observed for the control and pure BNC samples. By day 16, the control sample reached a value of 48.1 ± 2. In contrast, chicken breasts treated with JPs, especially at 0.03%, had significantly higher (*P* < 0.05) L* values than the control, attributed mainly to the pale yellow hue of the JPs. Similarly, Razavi et al.^24^ reported that meat samples containing 0.05% JPs showed the highest L* value. The a* (redness) and b* (yellowness) values fluctuated significantly. By day 16, both a* and b* values increased in the control sample. However, there was no notable difference in a* values between the sample containing 0.03% JPs and the control. The BNC-JPs_0.03%_ samples displayed a lower b* value than the control, and the pure BNC recorded the lowest b* among all samples.

**Table 2:** The changes in the color (L*, a*, and b*) of chicken breast meat covered with BNC- JPs films during refrigerator storage.

	Treatments					
	Days	Control	BNC	BNC-JPs _0.01%_	BNC-JPs _0.02%_	BNC-JPs _0.03%_
L*	0	52.5 ± 1.5^a^	52.5 ± 1.5^a^	52.5 ± 1.5^a^	52.5 ± 1.5^a^	52.5 ± 1.5^a^
	4	49.2 ± 0.9^c^	50.3 ± 2^c^	56.4 ± 1.5^b^	65.6 ± 1.8^a^	68.7 ± 0.5^a^
	8	50.9 ± 1^c^	54.0 ± 1.6^c^	65.0 ± 1.5^b^	64.5 ± 1.2^b^	71.5 ± 0.8^a^
	12	46.3 ± 1.5^d^	43.7 ± 0.5^d^	53.7 ± 1.3^c^	65.9 ± 1.5^b^	71.1 ± 0.9^a^
	16	48.1 ± 2^c^	51.1 ± 1.2^c^	63.5 ± 1.8^b^	66.0 ± 0.5^ab^	68.7 ± 1.5^a^
a*	0	− 3.1 ± 1^a^	− 3.8 ± 1^a^	− 3.08 ± 1^a^	− 3.08 ± 1^a^	− 3.08 ± 1^a^
	4	− 0.7 ± 0.4^b^	− 1.2 ± 0.5^ab^	− 2.6 ± 0.5^ab^	− 2.9 ± 0.8^a^	− 2.9 ± 1.2^a^
	8	− 2.4 ± 0.9^a^	− 2.7 ± 0.8^a^	− 3.6 ± 0.6^a^	− 2.8± 0.5^a^	− 3.8 ± 0.5^a^
	12	− 3.6 ± 0.5^a^	− 0.7 ± 0.5^b^	− 3.2 ± 0.8^a^	− 3.7 ± 1^a^	− 3.5 ± 0.7^a^
	16	− 4.0 ± 0.5^a^	− 1.3 ± 0.8^b^	− 3.0 ± 1.3^ab^	− 3.4 ± 0.5^ab^	− 3.5 ± 0.5^a^
b*	0	10.1 ± 0.5^a^	10.1 ± 0.5^a^	10.1 ± 0.5^a^	10.1 ± 0.5^a^	10.1 ± 0.5^a^
	4	7.5 ± 2.2^b^	10.0 ± 0.5^ab^	12.7 ± 2.1^a^	13.9 ± 1^a^	12.8 ± 0.9^a^
	8	13.2 ± 1.7^ab^	16.0 ± 0.7^a^	12.1 ± 1.5^b^	13.5 ± 1.8^ab^	12.5 ± 1.5^ab^
	12	13.6 ± 1.5^a^	11.2 ± 1.6^ab^	12.8 ± 1.5^ab^	11.0 ± 2.3^ab^	8.7 ± 1.4^b^
	16	15.0 ± 0.9^a^	11.1 ± 1.5^b^	16.02 ± 1^a^	14.9 ± 1.5^a^	13.16 ± 0.8^ab^

Within each row, values with different letters are significantly different (*P* < 0.05).

## Conclusion

The objective of this research was to develop an antibacterial and antioxidant film based on JPs and BNC for use as active packaging to minimize the public health risks associated with *S*. Typhimurium contamination in poultry products. MTT assays revealed that JPs were only slightly toxic to human gastric cancer cells, suggesting their possible suitability for food-related applications. Furthermore, the effective incorporation of JPs within the BNC matrix was verified using FTIR analysis. BNC-JPs films showed excellent antimicrobial and antioxidant properties, which depended on the JPs concentration used. The films significantly reduced the populations of *S*. Typhimurium and mesophilic bacteria in chicken breast meat during storage under chilled conditions. Moreover, compared with unwrapped samples and samples wrapped with pure BNC film, the BNC-JPs films showed improvements in meat quality parameters, including pH, TVB-N, and TBARS values. These findings highlight the potential of bio-based, biodegradable BNC-JPs films with strong antimicrobial properties for use within the poultry industry. While these findings demonstrate promising antimicrobial performance of BNC-JPs films, challenges remain on large-scale optimization of JP synthesis, long-term stability under diverse storage conditions, and regulatory approval for food-contact applications.

## Data Availability

The authors declare that data is provided within the manuscript file.
